# The Treatment of Recurrent Urothelial Tumors of the Upper Urinary System and at Urostomy Site following Radical Cystectomy with Intraureteral Bacillus Calmette-Guérin and Cryotherapy

**DOI:** 10.1155/2013/490373

**Published:** 2013-03-14

**Authors:** Abdullah Demirtaş, Yunus Emre Yıldırım, Ayten Ferahbaş, Emre Can Akınsal, Oguz Ekmekçioğlu, Atila Tatlışen

**Affiliations:** ^1^Department of Urology, Erciyes University Medical Faculty, 38039 Kayseri, Turkey; ^2^Department of Dermatology, Erciyes University Medical Faculty, 38039 Kayseri, Turkey

## Abstract

Urinary bladder carcinoma is the second most common cancer of the urinary system. The recurrence rate in the upper urinary system (UUS) for urothelial cancers is around 3% following radical cystectomy. The followup generally consists of imaging studies and urinary cytology, although there are no prospective data on the frequency, the mode, and the duration of followup. In patients carefully selected according to risk factors, kidney-sparing minimally invasive methods (ureteroscopic procedures, percutaneous approach, and local drug instillation) appear as contemporary alternatives for low-grade and low-stage primary UUS. In this paper, we present the patient who underwent radical cystectomy with urinary diversion ureterocutaneostomy, was diagnosed with widespread bilateral UUS tumors and recurrent tumor at the urostomy site at active followup, for which he was given local Bacillus Calmette-Guérin (BCG) and cryotherapy, and was followed by disease-free for 2 years thereafter.

## 1. Introduction

Urinary bladder cancer is the second most common cancer of the urinary system and is responsible from 3% to 5% of all cancer-related mortality [1]. More than 100,000 cases annually are diagnosed with advanced-stage bladder cancer worldwide [2]. 

Radical cystectomy, which is considered the most effective treatment option for invasive bladder cancer, is curative in only half of the patients. The remaining local recurrences are common, the majority of which are distant metastases. Most of these recurrences appear in the first 2 years, following surgery [3]. The rate of recurrence of urothelial cancers in the upper urinary system (UUS) is around 3% after radical cystectomy [4]. The main objective of the UUS followup following cystectomy is to diagnose recurrent cancer before it metastasizes locally or distantly and at an as early as possible to allow for treatment. Thus, the diagnosis of recurrent cancer is of paramount importance before the clinical symptoms appear [5]. Followup usually consists of imaging studies and urinary cytology, although there are no prospective data on the frequency, the mode, and the duration of followup. 

In this paper, we present the patient who underwent radical cystectomy ureterocutaneostomy, was diagnosed with widespread bilateral UUS tumor and recurrent tumor at the urostomy site at active followup, for which he was given local Bacillus Calmette-Guérin (BCG) and cryotherapy, and was followed disease-free for 2 years thereafter. 

## 2. A Case Report

**Figure 1:**
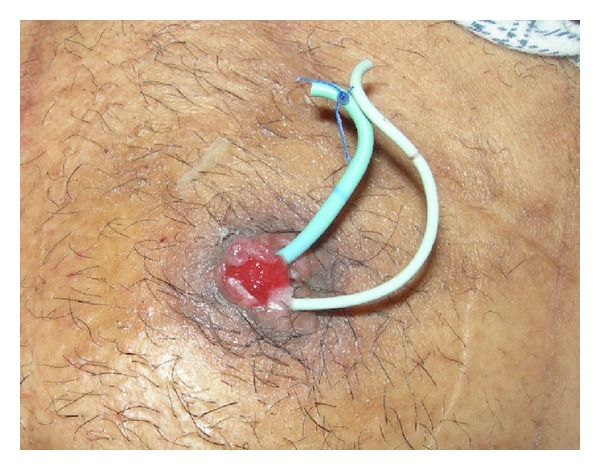
View of the ostomy before cryotherapy.

A 54-year-old male patient underwent radical cystoprostatectomy, ureterocutaneostomy (patient preference), and bilateral iliac lymph node dissection following a positive test result of transurethral resection-bladder (TUR-B) pathology for a low-grade urothelial carcinoma with muscle invasion. Perioperative ureter lower end frozen examination revealed no tumor at the lower ends of both ureters. The tumor's pathological stage was pT2N0M0 (urothelial carcinoma, high grade, positive for muscle invasion, and bilateral (right: 11, left: 7) reactive lymph nodes). He began and routine followup after the operation. Urinary cytology was class 2 at the postoperative 3rd-month control and class 4 at the 6th month; his urinary cytology was repeated, and the test result was reported as class 5. Furthermore, his physical examination revealed a hyperemic and edematous area that was tuberous at the site of the urostomy (Figure 1). The patient was asymptomatic. No pathology was evident in imaging examinations.

**Figure 2:**
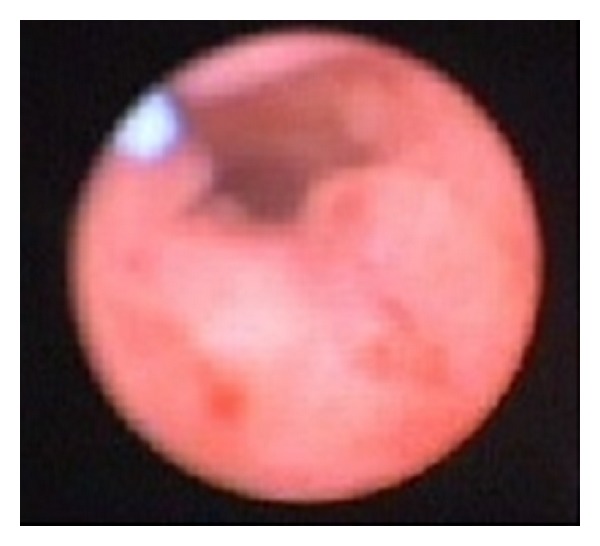
View of the ureter before intraurethral BCG treatment.

An excisional biopsy was taken from the suspicious hyperemic lesion at the urostomy site. Bilateral ureterorenoscopy was performed. Multiple tumors smaller than 0.5 cm were detected in both ureters, and a punch biopsy was taken (Figure 2). 

The pathological examination of the sample tissues revealed urothelial carcinoma, cutaneostomy skin excisional biopsy, in situ carcinoma right, and left suspicious ureteral lesions. A local therapy was planned as he had bilateral ureteric recurrences that involved the urostomy site, and the patient preferred the local therapy. With these findings, he was given BCG 81 mg diluted in a 50 cc isotonic saline solution and given in 25 cc to the right and left ureters each from a height of 40 cm as intraureteral chemotherapy every second week for a total of 3 courses. A dermatology consultation was obtained for the tumoral lesion at the cutaneostomy, and 4 courses of cryotherapy with 21-day intervals were conducted on this site as the initial therapy. 

Cryotherapy is a technique that employs intensely low temperatures to eliminate the diseased tissues. For this purpose, solid carbon dioxide (dry ice) at −78.5°C or liquid nitrogen at temperatures up to −190°C are used. The gas that is to be used in the freezing process is passed through the probe and causes the probe to cool excessively. When the gas used in the cooling process is pushed toward the end of the probe, the volume of the gas instantly expands, and as it absorbs heat from the environment, the probe end is cooled. The cell death is provided by the transformation of the pure water in and out of the cell into ice crystals. 

**Figure 3:**
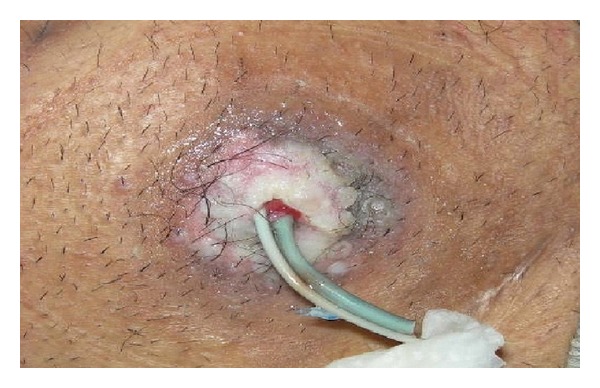
View of the ostomy following cryotherapy.

According to these principles, the patient underwent 4 courses of cryotherapy of 30–70 seconds with a double cooling-thawing cycle, and it was observed that the tumoral lesions at the cutaneostomy site regressed to skin level. At the mid-term evaluation, it was decided to continue the cryotherapy. After observing that the patient's lesion completely recovered as a result of 9 courses of cryotherapy, a few punch biopsies were taken from the cutaneostomy region (Figure 3). 

**Figure 4:**
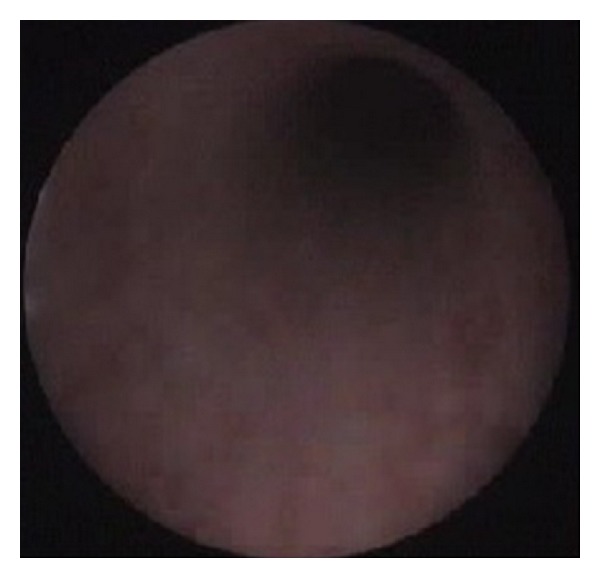
View of the ureter before intraurethral BCG treatment.

The pathological examination of the punch biopsies was negative for tumoral lesions. In addition, urinary cytology was observed as class 2, and the flexible ureterorenoscopy was also negative for a tumoral presence on both sides of the UUS. The patient was enrolled in active followup. His later urinary cytological examinations were also consistent with class 2, and endoscopies were negative for tumors (Figure 4). He has been at active followup for 2 years with no urological symptoms or progression.

## 3. Discussion

Survival after recurrences in the UUS following radical cystectomy is generally disappointing. The rate of survival for more than 3 years has been reported as 0%–25%. Thus, the main priority of the UUS followup after cystectomy is to diagnose the recurrences as early as possible to allow for an effective treatment. Diagnosis of the cancer recurrence before the symptoms develop is very important. Followup generally consists of urinary cytology and imaging modalities. One of the controversial topics regarding cytology, which is commonly used for followup following radical cystectomy, is the positivity of cytology before any other positive finding appears. There is limited information in the literature regarding this issue. Raj et al. detected uroepitheleal recurrence at a median of 2.1 years after surgery in 57 (56.4%) of 101 patients [6]. Patients in whom the only positive finding is a positive cytology must be monitored carefully for an extended period. However, although strict followup protocols are followed UUS tumor recurrences in the majority of patients cannot be detected at an early and asymptomatic stage when the tumor load is low, and thus the chance for cure is relatively higher. Diagnosis in those patients can only be made from symptoms such as hematuria, pain, infections, and weight loss that appear as a result of clinical progression at a later phase.

The other modality in oncological followup is imaging which is usually applied in the form of intravenous urography (IVU) or computerized tomography (CT). IVU and CT can only diagnose 0%–55% of UUS recurrences. Although no method is superior to another in the diagnosis of the UUS tumors, CT has the advantage of showing retroperitoneal lesions, and organ recurrences better. The diagnostic power of CT is related with the localization of the lesion at UUS and its size. For example, its diagnostic power is around 78%–94% in lesions located at the renal pelvis, whereas it is only 17%–53% in ureteric localizations. The chance of detecting small (<2 mm) lesions by CT is also low [7]. As the lesions in our case were smaller than 0.5 cm and located at the lower ends of the ureters, the imaging modality could have missed them.

By the very nature of urothelial cancer, all local and distant recurrences other than “new” tumors originating from the remaining uroepithelium following radical cystectomy are considered systemic disease. The only current treatment modality that has been shown to boost survival in UUS recurrences is nephroureterectomy. Regional lymph node involvement has been reported at between 32% and 83% in those undergoing nephroureterectomy for UUS recurrence after cystectomy. Adjuvant chemotherapy application also has limited benefit following lymph node dissection and nephroureterectomy in patients with lymph node involvement. Unfortunately, the long-term disease-free survival rate is below 5% in those patients. Thus, kidney-sparing minimally invasive methods (ureteroscopic interventions, percutaneous approach, and local drug instillation) seem to be current alternatives for low-grade, low-stage primary UUS tumors in patients selected carefully based on risk factors [8].

After minimally invasive or nephroureterectomy therapies, the actual main determinants of prognosis are the stage, grade, localization, and size of the tumor. One of the most important factors determining the prognosis in UUS transitional cell carcinomas is the tumor grade [9]. Large high-grade tumors with an advanced stage have worse prognosis. According to these criteria, at least 75% of UUS recurrences following cystectomy carry bad prognostic signs, and, therefore, they put patients at high risk for progression. Furthermore, recurrent UUS cancers have a worse prognosis than primary UUS tumors even after tumor grade and stage are controlled. In addition, tumor localization is also an important factor for survival. It has been reported that recurrences at the ureteroileal anastomosis line have a higher potential to progress rapidly and metastasize than recurrences at a distance from the anastomosis site [10]. We employed BCG instillation to both ureters and local cryotherapy to the ostomy site given that the recurrences were at both ureters, involved the urostomy site, and the patient preferred local therapy. 

In conclusion, recurrent UUS urothelial tumors following radical cystectomy can be treated by systemic and local therapies in cases in which surgical therapy is not an option. The localization, grade, and size of the recurrent tumor should certainly be taken into consideration in that decision. In addition, alternative treatment modalities such as locally-administered drug instillation (BCG) and cryotherapy should be considered in localized recurrent urinary system urothelial tumors.
